# Role of the Receptor for Advanced Glycation End Products (RAGE) and Its Ligands in Inflammatory Responses

**DOI:** 10.3390/biom14121550

**Published:** 2024-12-04

**Authors:** Kaylen Cross, Stefan W. Vetter, Yousuf Alam, Md. Zahidul Hasan, Anupom Deb Nath, Estelle Leclerc

**Affiliations:** Department of Pharmaceutical Sciences, North Dakota State University, Fargo, ND 58105, USA; kaylen.cross@ndsu.edu (K.C.); stefan.vetter@ndsu.edu (S.W.V.); yousuf.alam@ndsu.edu (Y.A.); mdzahidul.hasan@ndsu.edu (M.Z.H.); anupom.nath@ndsu.edu (A.D.N.)

**Keywords:** RAGE, inflammation, S100, DAMP, stress

## Abstract

Since its discovery in 1992, the receptor for advanced glycation end products (RAGE) has emerged as a key receptor in many pathological conditions, especially in inflammatory conditions. RAGE is expressed by most, if not all, immune cells and can be activated by many ligands. One characteristic of RAGE is that its ligands are structurally very diverse and belong to different classes of molecules, making RAGE a promiscuous receptor. Many of RAGE ligands are damaged associated molecular patterns (DAMPs) that are released by cells under inflammatory conditions. Although RAGE has been at the center of a lot of research in the past three decades, a clear understanding of the mechanisms of RAGE activation by its ligands is still missing. In this review, we summarize the current knowledge of the role of RAGE and its ligands in inflammation.

## 1. Introduction

The receptor for advanced glycation end products (RAGE) is a multi-ligand cell surface receptor and a member of the immunoglobulin superfamily. RAGE is constitutively expressed at high levels during the early developmental stages, but its expression is low in most adult cell types, including endothelial cells, epithelial cells, neurons, cardiomyocytes, vascular smooth muscle cells, and immune cells (neutrophils, monocytes/macrophages, lymphocytes, and dendritic cells (DCs). One exception is the lung tissue, where RAGE expression is high [1]. The exact reason for the high expression of RAGE in the lungs is unknown, but recent studies suggest that RAGE is an important mediator in many pulmonary inflammatory disorders such as asthma, pulmonary fibrosis, chronic obstructive pulmonary disease, cystic fibrosis, or allergic airway inflammation [2,3].

Early experimental evidence in support of the role of RAGE in inflammation was reported by Hofmann et al. in 1999 [4]. Using a combination of in vitro and in vivo experiments, the authors showed that RAGE activation by its ligand S100A12 (also called EN-RAGE) stimulated several types of immune cells and increased the migration of mononuclear phagocytes, the secretion of pro-inflammatory cytokines (IL-1β and TNF-α) by macrophages, and the proliferation of peripheral blood mononuclear cells (PBMC) [4]. The authors further confirmed the role of RAGE in inflammation using mouse models of delayed-type hypersensitivity and colitis [4]. Soon after these important studies, RAGE was recognized as a key contributor in inflammatory processes [5,6].

One characteristic of RAGE is that it is a promiscuous receptor. RAGE can be activated by many structurally different ligands, in cell-type-specific manners. The list of RAGE ligands increases frequently and includes diverse glycated proteins (β2 microglobulin, collagen, serum albumin, etc.), multimeric and aggregated forms of peptides (amyloid β peptides, transthyretin (TTR), islet amyloid polypeptide), small α-helical proteins (S100s, high-mobility group box 1 (HMGB1)), transmembrane proteins (MAC-1), nucleic acids, C1 q, phosphatidylserine, and lysophosphatidic acid [7,8,9,10,11,12,13,14,15,16,17]. We will review here the effects of the RAGE ligands on immune cells that are most relevant to inflammation, and give examples of the known roles of RAGE in inflammatory disorders.

## 2. RAGE Structure and Isoforms

**Figure 1:**
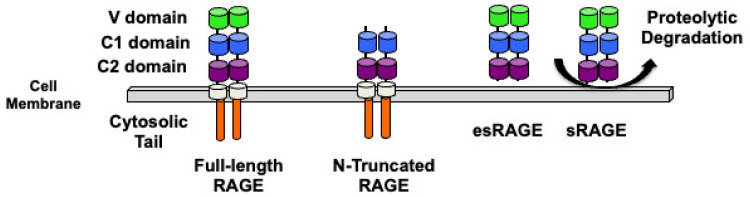
RAGE is a transmembrane protein with an extracellular part consisting of three Ig-fold domains, a variable-type (V-domain) and two constant-type domains (C1 and C2), a single-pass transmembrane domain, and a cytosolic tail. The most common RAGE isoform is full-length RAGE. Other important isoforms are the N-truncated form and the two soluble forms of the receptor ectodomain. Endogenously secreted esRAGE is a splice variant of RAGE. sRAGE is generated by proteolytic clipping following the activation of membrane proteases.

RAGE consists of an extracellular part, a single-pass transmembrane domain, and a short intracellular portion (Figure 1) [18]. RAGE contains an N-terminal signal peptide (residues 1–22) that is cleaved and absent in the mature form of the protein (residues 23–404) [19,20]. The signal peptide facilitates the insertion of the receptor into the endoplasmic reticulum and trafficking through the Golgi towards the plasma membrane [21]. As a result, RAGE is found expressed at the surface of many cell types, such as endothelial cells [22] and lung alveolar cells [23], but also immune cells, including T and B lymphocytes and macrophages [24].

The extracellular part of RAGE is the site of ligand binding and consists of three Ig-domains: a variable-like (V) domain, a constant type 1 (C1) domain, and a constant type 2 (C2) domain (Figure 1). Although some ligands do bind to the C1 or C2 domain, the V domain appears to be the primary site of ligand binding [13,25]. The transmembrane segment connects the extracellular and intracellular portions of the receptor (Figure 1) [9]. The intracellular segment is a short, highly charged cytosolic tail that binds to adaptor proteins, including diaphanous-related formin 1 (DIAPH 1) and toll-interleukin 1 receptor domain-containing adaptor protein (TIRAP), mediating downstream cell signaling (Figure 1) [26,27,28].

Multiple spliced isoforms of RAGE have been described in human cells and tissues [29,30]. The main isoform of RAGE is membrane-embedded full-length RAGE that is mostly present at the surface of cells, but can also occur intracellularly (Figure 1) [31]. One isoform lacking the V domain (N-truncated RAGE), and thus unable to bind to most ligands, has been described in endothelial cells [30]. Two other important RAGE isoforms are secreted, and are referred to as soluble RAGE, because they lack the transmembrane domain and the cytosolic tail. The first type of soluble form, sRAGE, results from proteolytic cleavage of membrane-bound, full-length RAGE by matrix metalloproteinases and ADAM10 [32,33]. The second form of soluble RAGE, which is referred to as endogenously secreted (es)RAGE, is the result of alternative splicing [30,34]. Both secreted forms of RAGE contain ligand binding domains and therefore bind to RAGE ligands (Figure 1).

Since the identification of soluble forms of RAGE, many studies have investigated the role of sRAGE and esRAGE in relation to full-length RAGE in many human diseases, including cardiovascular disease, neurodegenerative disorders, and inflammatory disorders [35,36,37] (reviewed in [38]). When used as a pharmacological agent, both in in vitro and mouse models of disease, sRAGE successfully prevents the activation of RAGE, either by interacting with and sequestering circulating RAGE ligands, or by forming a hybrid sRAGE/RAGE complex lacking the ability to initiate cellular signaling [9]. However, when comparing the levels of circulating sRAGE and esRAGE in many human diseases, there is currently no clear consensus on the role of these isoforms in pathologies or as biomarkers, and many questions remain unanswered, as pointed out by Schmid in her excellent review [38].

## 3. Signaling Pathways Activated Following RAGE Interaction with Its Ligands

**Figure 2:**
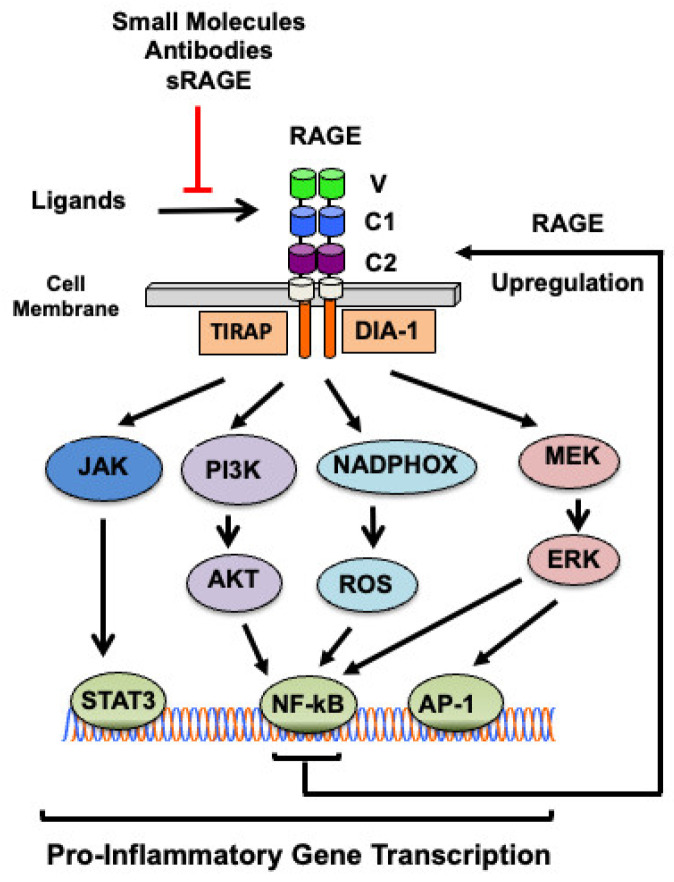
Overview of the RAGE-dependent signaling pathways. Binding of ligands to RAGE leads to the activation of MEK/ERK, PI3K/AKT, or JAK, or the activation of NADPH oxidase (NADPHOX) with the resulting production of reactive oxygen species (ROS), leading to the translocation and activation of several transcription factors (NF-κB, AP-1, STAT3) that promote the transcription of pro-inflammatory genes. RAGE activation results in RAGE upregulation through a NF-κB-dependent positive feedback loop, because of the presence of functional NF-κB binding sites in the RAGE promotor [39,44]. Most RAGE ligands bind to the V domain of RAGE. RAGE activation by its ligands can be inhibited by the soluble form of the receptor, anti-RAGE antibodies, and small molecule inhibitors.

The interaction of RAGE with its ligands results in the activation of several signaling pathways, including the phosphoinositide 3-kinase/Ak strain transforming kinase (PI3K/AKT), mitogen-activated protein kinase/extracellular signal-regulated kinase (MAPK/ERK), and janus kinase/signal transducer and activator of transcription (JAK/STAT) pathways (Figure 2) [39,40,41,42,43]. The two adaptor proteins TIRAP and DIA-1 transmit the signal from RAGE to the downstream key signaling molecules (Figure 2) [26,27,28]. The activation of these different signaling pathways results in the activation of transcription factors such as nuclear factor kappa B (NF-κB), specific protein 1 (SP1), and activating protein-1 (AP-1), further amplifying the cellular response (Figure 2) [42,44,45,46,47,48,49,50,51,52,53,54,55,56,57,58,59]. Because NF-κB response elements are present in the promoter region of the RAGE gene, a consequence of RAGE activation by its ligands is the upregulation of RAGE itself [39], as well as the expression of pro-inflammatory cytokines (Figure 2) [26,60].

## 4. RAGE Ligands and Their Effects on Immune Cells

As mentioned in the introduction, one particularity of RAGE is that it can be activated by many structurally and functionally diverse ligands. The current list of RAGE ligands includes advanced glycation end products (AGEs), high-mobility group box 1 (HMGB1) protein, S100 proteins, lysophosphatidic acid, amyloid β, phosphatidylserine, complement protein C1q, transthyretin (TTR), islet amyloid polypeptide (IAPP), and nucleic acids [7,8,9,10,11,12,13,14,15,16,17]. In the following paragraphs, we will discuss the effect of inflammation-relevant RAGE ligands on immune cells (summarized in Table 1).

### 4.1. Advanced Glycation End Products (AGE)

The ligands that gave the name to the receptor are AGEs. AGEs are mostly generated during the Maillard reaction, a process involving the non-enzymatic reaction between reactive carbonyl groups, present in reducing carbohydrate molecules and primary amino groups present on proteins, lipids, and nucleic acids [20,94,95]. Different types of AGEs have been identified depending on their precursor carbonyl compound: they include glyoxal-derived AGEs, methyglyoxal-derived AGEs, glyceraldehyde-derived AGEs, and fructose- or glucose-derived AGEs [95].

AGEs can contribute to pathological processes by altering protein structure via crosslinking. An example is glycated collagen. In elderly patients, glycated collagen is responsible in part for increased stiffness of collagen networks in the bone, thereby increasing the risk of bone fracture [96]. Hemoglobin (Hb) A1C is another example of glycated protein. It is found in elevated levels in the blood of diabetic patients and serves as clinical indicator of diabetes [97,98].

Besides directly modifying protein structure and function, AGEs can promote inflammation through the activation of diverse cell surface receptors, such as scavenger receptors (SR) type A-I/II and B-I, CD36, galactin-3, and RAGE [99,100,101,102,103,104,105,106,107]. In an early study, Miyata et al. showed that the interaction of AGE-modified β2-microglobumin with RAGE resulted in increased production of TNF-α and of oxidative stress in mononuclear phagocytes (Table 1) [61]. AGE-ovalbumin has been shown to enhance RAGE expression, NF-κB activation, and IL-6 expression in immature human dendritic cells (DC) [62]. In a different study, in addition to enhancing RAGE expression, AGE-BSA was shown to stimulate the maturation of monocyte-derived DCs and to enhance the capacity of these cells to activate T cells [63]. AGE-BSA can also stimulate exocytosis of mast cells, resulting in histamine release and ROS production [64]. There is also evidence that AGEs, produced by neutrophils, promote phosphorylation of receptor-interacting protein kinase 3 (RIPK3), an important initiator of myocardial necroptosis, via RAGE, exacerbating myocardial infarction and other related conditions (Table 1) [108].

Following their interaction with RAGE, AGEs can be internalized. Early studies by Schmidt et al. showed that AGE albumin labeled with gold particles decorated cellular structures resembling endosomes [109]. A later study by Sevillano et al. showed that CHO-K1 and Neuro-2 cells, overexpressing RAGE, responded to AGE stimulation by increasing ERK and NF-κB activation [110]. Interestingly, the authors showed that AGE-dependent activation of ERK and NF-κB could be blocked using inhibitors of internalization [110], an indication that RAGE can facilitate the transport of RAGE ligands inside cells.

Although we will not expand on the role of RAGE and its ligands in cancer in this review, AGEs can be generated in large amounts in cancer cells, thereby contributing to an inflammatory tumor microenvironment through the activation of RAGE- and NF-κB-dependent transcription of pro-inflammatory mediators [111]. Indeed, two main precursors of AGEs, methylglyoxal, and glyceraldehyde, can be generated as byproducts of glycolysis. Glycolysis is an essential metabolic process in cells, allowing them to utilize glucose as a source of energy [112]. Cancer cells are highly proliferative and thus have a higher requirement for ATP than non-cancer cells. Cancer cells often use glycolysis to generate ATP, a process referred to as the Warburg effect [113]. Because of enhanced glycolytic flux, cancer cells generate higher levels of methylglyoxal and glyceraldehyde, thus resulting in higher levels of AGEs [114].

### 4.2. High-Mobility Group Box 1 (HMGB1) Protein

HMGB1 was the first non-AGE RAGE ligand identified [68] and was named for its high mobility during gel electrophoresis [115]. HMGB1 is a multifunctional protein that possesses nuclear, intracellular, and extracellular functions [116]. Nuclear HMGB1 binds to chromatin-associated DNA and serves as a DNA chaperone. It also modulates gene transcription by interacting directly with DNA regulatory regions and facilitating the recruitment of transcription factors [116]. The main function of cytoplasmic HMGB1 is to modulate autophagy by associating with beclin-1 [117], and to participate in the regulation of inflammasomes [118,119]. When released in the extracellular space, HMGB1 acts as a damage-associated molecular pattern (DAMP) with cytokine activities leading to multiple cellular responses, such as cell differentiation, cell migration, tissue regeneration, angiogenesis, bacterial killing, cell proliferation and cell death, senescence, and cellular inflammation [116]. Multiple cell surface receptors for HMGB1 have been identified and include RAGE, Toll-like receptors (-2; -4 and -9), CXCR4, and IL-1-R [116]. Among these receptors, RAGE and TLR-4 have been the most thoroughly studied [120].

Structurally, HMGB1 consists of three functional regions, including two N-terminal DNA-binding structural domains (A-box (aa 9–79) and B-box (aa 95–163)), and an acidic C-terminal domain (aa 186–215) [121]. The protein contains three redox-sensitive cysteine residues at positions 23, 45, and 106 [122], and the function of HMGB1 depends on the redox state of these cysteine residues. For example, the presence of a disulfide bridge between Cys 23 and Cys 45, and of a thiol group at position 106, is essential for the cytokine-like function of HMGB1 via interaction with the Toll-Like Receptor (TLR) 4/MD2 complex [122]. On the other hand, when all three cysteine residues are fully reduced, HMGB1 exerts chemotaxis activity through the formation of a complex with CXCL12 and the activation of CXCR4 [122]. HMGB1 also contains two lysine-rich nuclear localization sites, one located in Box A (aa 28–44), and the second in Box B (aa 179–185). Hyperacetylation of these lysine residues results in the relocation of HMGB1 from the nuclear compartment to the cytoplasm, thereby enhancing its accumulation in secretary lysosomes, and facilitating its later secretion into the extracellular medium, as shown in monocytes and macrophages [123]. In addition, hyperacetylation of HMGB1 prevents its re-entry into the nucleus [120].

A study by Huttunen et al. showed that RAGE interacted with aa 150–183 of HMGB1, resulting in increased neurite outgrowth in vitro and increased formation of lung metastases in a mouse model of melanoma [52,67]. A different study identified amino acids 23–50 of HMGB1 as a second region interacting with RAGE and being responsible for reversing apoptosis-induced tolerance in dendritic cells [124], demonstrating multiple binding motifs for RAGE within HMGB1.

Both active and passive release mechanisms of HMGB1 into the extracellular medium have been proposed [120,125]. For example, HMGB1 was shown to be secreted by lysosome exocytosis in activated monocytes [126]. In a different study, Lamkanfi et al. showed that the secretion of HMGB1 from LPS-stimulated murine bone-marrow-derived macrophages depended on the activation of the inflammasome and caspase 1 [127]. In addition, the secretion of HMGB1 can be triggered by a variety of molecules, including cytokines, ROS, and changes in calcium concentration, as well as by cell–cell interaction [125]. The post-translational state (i.e., oxidative state of cysteine residues and acetylation of lysine residues) of HMGB1 can also influence its release mechanisms [120,125].

Once secreted outside the cells, HMGB1 acts alone or in complex with other ligands [128]. Qin et al. showed that recombinant HMGB1 was devoid of pro-inflammatory activity on murine peritoneal macrophages unless it was in a complex with LPS [65]. HMGB1/LPS complexes were shown to signal through RAGE, resulting in p38- and NF-κB-activation (Table 1) [65]. The study of Sha et al. also showed that only when HMGB1 was isolated from cultured cells in the presence of IL-1β, interferon gamma (INF-γ), and TNF-α was it able to activate murine macrophages and neutrophils, whereas HMGB1 alone was not able to stimulate the expression of pro-inflammatory cytokines by these immune cells (Table 1) [129]. There is evidence that RAGE facilitates the internalization of HMGB1 in macrophages and monocytes, resulting in lysosome rupture, cathepsin 1, and caspase 1 activation in a programmed cell death called pyroptosis [68]. HMGB1-/RAGE-dependent endosomal internalization and pyroptosis has also been suggested in endothelial cells [130] and macrophages [70]. In this latter study, Deng et al. showed that HMGB1, that was secreted by LPS-stimulated hepatocytes, formed a complex with LPS at the surface of macrophages, before being internalized by macrophage-expressed RAGE [70]. Following HMGB1/LPS complex internalization in endosomes and later in lysosomes, the authors showed that HMGB1 was able to destabilize the membranes of the lysosomes, resulting in the leakage of LPS into the cytoplasm [70]. Once in the cytoplasm, Deng et al. showed that LPS could activate caspase 11, leading to pyroptosis [70], in agreement with the data from Xu et al. [68]. HMGB1 binds to nucleic acids [131] and HMGB1 was suggested to serve as a nucleic acid delivery cargo to intracellular TLR-9 (Table 1) [66,132,133]. The role of HMGB1/RAGE as co-transporter of molecules has most recently been demonstrated by Chang et al., where the RAGE/HMGB1 complex was shown to facilitate the transport of a bacterial toxin to other intracellular receptors [134]. These data further suggest the emerging function of RAGE as cargo for the delivery or internalization of other molecules in cells.

### 4.3. Nucleic Acid

RAGE binds to both DNA and RNA (Table 1) [17,135]. The study of Sirois et al. showed that the binding of DNA to RAGE triggers the internalization of the RAGE/DNA complex, resulting in the delivery of the nucleic acid to intracellular TLR-9 [17,135]. Liu et al. showed that HMGB1/DNA complexes stimulated the activation of the inflammasome marker AIM2, as well as the production of the inflammasome-dependent early pro-inflammatory cytokine IL-1β, in THP-1 and HL-60 human monocytes in culture, in a RAGE-dependent manner (Table 1) [71]. Surprisingly, the authors also showed that HMGB1/DNA complexes could stimulate autophagy-related gene 5 (ATG5)-dependent autophagy, in a RAGE-dependent manner, in these cells, suggesting that HMGB1/DNA complexes, in coordination with RAGE, participate in both the initiation and termination of inflammatory signals in monocytes (Table 1) [71]. Recently, nuclear DNA in complex with HMGB1 was shown to trigger cell death in murine RAW264.7 macrophages, through the activation of AKT and the release of TNF-*α* in a RAGE-dependent manner [72].

### 4.4. MAC-1

Another immunologically relevant ligand of RAGE is MAC-1. MAC-1 is a member of the β2 integrin subfamily [136,137]. This family consists of four members. Each member is composed of a β subunit (in this case β2 CD18), and a subunit that can be of four subtypes (L (CD11a), M (CD11b), X (CD11c), or D (CD11d)). The β2 integrin subfamily plays crucial roles in the immune system by modulating adhesion between cells and components of the extracellular matrix (ECM), cytoskeletal reorganization, cell signaling, or opsonization of complement-coated pathogens (reviewed in [137]). The β2 integrin subfamily member Cd11b/CD18, also called MAC-1, was shown to stimulate neutrophil migration through synovial and dermal fibroblast barriers [136] and to modulate T cell function when expressed at the surface of dendritic cells [138]. MAC-1 interacts with RAGE, and this interaction results in enhanced leukocyte recruitment [75]. MAC-1 also interacts with the RAGE ligand HMGB1, and colocalization between RAGE and MAC-1 was shown to be enhanced by HMGB1 in a monocytic cell line [76,137]. In addition, neutrophil recruitment by RAGE/MAC-1 was enhanced by HMGB1 [76]. Interestingly, the binding of S100B to RAGE was also shown to enhance RAGE/MAC-1 interaction [75], suggesting a complex regulation of RAGE/MAC-1 by the alarmins S100B and HMGB1 in inflammatory conditions.

### 4.5. C1q

The C1q complement protein has also been shown to interact with RAGE and to enhance cell adhesion and phagocytosis (Table 1) [14]. In an immunoprecipitation study, it was shown that both RAGE and MAC-1 bound to C1q, and that the pull-down of RAGE and C1q increased when these two proteins were co-incubated [14]. During the classical complement activation pathway, C1q helps in the elimination of microbes by phagocytosis. Another important function of C1q is in the clearance of apoptotic cells during an adaptive immune response [139]. In this case, C1q can opsonize the targeted apoptotic cells either directly or indirectly through binding to an antibody [140]. Strong evidence for the role of RAGE and MAC-1 in apoptotic cell removal was presented by Ma et al. [14], who, in agreement with earlier studies, suggested a role of RAGE in the removal of apoptotic cells or efferocytosis through interaction with the “eat me” signal phosphatidylserine [141,142,143].

C1q interacts with HMGB1, facilitating the formation of a multiprotein complex with RAGE and the leukocyte-associated Ig-like receptor-1 (LAIR-1) (Table 1) [73]. As a result of the complex formation, monocytes acquire an anti-inflammatory phenotype with the upregulation of anti-inflammatory cytokines such as IL-10, leading to resolution of inflammation [73]. In a follow-up study, the same research group further showed that the formation of the C1q/HMGB1 complex, in association with RAGE and LAIR-1, resulted in the expression of proresolving lipid mediators, such as lipoxin A4 and resolving D1 and D2, further supporting the role of this multiprotein complex in modulating the resolution of inflammation (Table 1) [74].

### 4.6. Phosphatidylserine

Phosphatidylserine (PS) is a phospholipid that is typically present in the inner layer of the membrane [144]. When cells undergo apoptosis, PS flips to the outer layer and is an important signal of recognition by macrophages [145]. Recent studies have suggested that the presence of PS on the outer layer of the plasma membrane also occurs in non-apoptotic forms of regulated inflammatory cell death, such as necroptosis [146]. PS binds to RAGE in vitro [141,142]. He et al. showed that RAGE-deficient alveolar macrophages were not able to phagocyte apoptotic thymocytes, whereas RAGE-positive alveolar macrophages could phagocyte the cells [142]. The RAGE dependent phagocytic activity of macrophages was confirmed by Friggeri et al. [141]. These authors also showed that pre-treatment of RAGE-positive macrophages with anti-RAGE antibodies led to reduced phagocytic activity. On the other hand, the overexpression of RAGE in HEK293 cells resulted in increased phagocytic activity [141]. Because the clearance of apoptotic cells by phagocytosis is an important step in the resolution of inflammation, these data demonstrate here again that RAGE not only participates in sustaining inflammation, but also in resolving inflammation.

### 4.7. Amyloid β Peptides

Amyloid *β* (A*β*) peptides are generated from the amyloid precursor protein (APP) through proteolysis by the *β*-site APP-cleaving enzyme 1 (BACE) and *γ* secretase [147]. We previously showed that both A*β* oligomers and fibrils bind to RAGE, albeit to different domains [13]. In the brain, the activation of RAGE by A*β* leads to oxidative stress through the activation of NADPH oxidase, and the production of pro-inflammatory cytokines such as TNF-*α*, IL-6, and macrophage colony-stimulating factor (M-CSF) by activated microglia (Table 1) [79]. Both neuronal cells and microglia have been shown to exhibit RAGE-dependent upregulation of macrophage colony-stimulating factor (M-CSF) [12,77]. In addition, in brain endothelial cells, RAGE has been shown to facilitate the transport of A*β* inside the brain [148]. Using a transgenic mouse model of Alzheimer’s disease expressing a mutant form of APP, and overexpressing RAGE, Fang et al. observed an increased production of the pro-inflammatory cytokines IL-1*β* and TNF-*α* in the brains of these mice compared to control mice [78]. In addition, the RAGE/mutant APP transgenic mice showed accelerated deterioration in spatial learning/memory abilities compared to their control littermates (Table 1) [78].

A*β* has also been shown to stimulate the expression of the T cell co-stimulatory CCR5 receptor in human brain microvascular endothelial cells (HBMEC), in a RAGE-dependent manner and through the activation of JNK, ERK, and PI3K, thereby facilitating the transendothelial migration of T cells [149].

Besides A*β*, RAGE has been shown to bind to other amyloid-forming proteins such as TTR [150] or IAPP [16]. RAGE-dependent activation of NF-kB has been observed in PC-12 cells overexpressing RAGE and stimulated with TTR [150]. Increased glycated TTR and RAGE protein levels have been found in patients suffering from rheumatoid arthritis, in association with increased levels of the pro-inflammatory cytokines IL-1*β*, IL-6, and TNF-*α*, suggesting a role of TTR/RAGE in this inflammatory disorder [151].

### 4.8. S100 Proteins

S100 proteins form another family of RAGE ligands. S100s are structurally very similar, and all contain one or two functional EF-Hand calcium binding sites [152]. Most S100 proteins form homodimers, but heterodimers and higher-order oligomers have also been observed [153]. S100 proteins have intracellular and extracellular functions and play important functions in health and diseases [154,155,156]. S100 proteins act as calcium sensors and transmit calcium signals in cells through their interactions with target proteins, resulting in the regulation of diverse cellular functions that include gene expression, cytoskeleton composition, cell cycle, and inflammatory responses [153]. The expression of S100 proteins is often tissue-specific, such as S100A1 in cardiac myocytes [157,158,159] or S100A3 in hair follicular cells [160,161,162]. When secreted into the extracellular medium, S100 proteins can act as DAMPs through their interaction with cell surface RAGE, resulting in NF-κB activation and the transcription and expression of pro-inflammatory cytokines (reviewed in [154,163]). Many S100 proteins are expressed by immune cells (lymphocytes, neutrophils, or macrophages) [154,164,165,166,167,168,169]. In the following paragraphs, we will summarize the recent knowledge on the role of specific S100 proteins in modulating immune cells.

Little is known about the role of S100A2 in inflammation, but S100A2-expressing transgenic mice were found to have a delayed response to cutaneous wound healing [170]. The role of S100A2 in inflammation is also suggested by the increased expression levels of S100A2 found in the skin of patients with atopic dermatitis and psoriasis [171]. Interestingly, a recent report described increased levels of S100A2 in the saliva of asthmatic patients [172], suggesting a role of this S100 protein in different types of inflammatory disorders (reviewed in [173]). S100A7 and S100A15 are also potentially involved in inflammatory skin diseases, as they have been found at higher levels in psoriatic plaques [174].

For a long time, S100A4 was considered to only play an important function in cancer [175]. Indeed, S100A4 interacts with and regulates many proteins of the cytoskeleton, resulting in changes in cellular morphology, adhesion, and motility [176]. As a result, S100A4 has been widely studied in the context of cancer, where it has been shown to stimulate cell migration and the formation of metastasis, hence its name of metastasin [175,177]. However, in recent years, increasing evidence has suggested that S100A4 also plays an important function in immune cells [178,179]. Through its interaction with cytoskeleton components, S100A4 can modulate the function of fibroblasts and endothelial cells, as well as the function of immune cells, including T lymphocytes, monocytes, macrophages, mast cells, dendritic cells, and neutrophils [80,180]. Evidence that S100A4 participates in inflammation is shown by the fact that mice devoid of S100A4 have defects in macrophage recruitment to the sites of inflammation, and macrophages derived from S100A4^−/−^ mice have defects in chemotaxis [80]. Extracellular S100A4 has been shown to attract immune cells, secrete cytokines, and participate in extracellular matrix remodeling processes [179]. S100A4 has also been shown to stimulate the production of the pro-inflammatory cytokines IL-1β, IL-6, and TNF-α from peripheral blood mononuclear cells (PBMC) isolated from patients with rheumatoid arthritis; however, these effects were not RAGE-dependent, but TLR-4-dependent (Table 1) [81]. Most recently, S100A4 was also shown to promote inflammation in the colon [181]. In this later study, the receptor that transmitted the effect of S100A4 was not determined [181]. Further studies would be necessary to determine the role of S100A4/RAGE and S100A4/TLR-4 on the modulation of immune cells.

Another member of the S100 protein family involved in inflammatory activities is S100A6. S100A6 is expressed in lymphocytes (reviewed in [182]) and has been shown to modulate host immune responses during infection with toxoplasma gondii [82]. In their study, Zhou et al. showed that the tgSAG1 protein from a parasite stimulated the production of TNF-α by human monocyte THP-1 cells in a S100A6/vimentin- and PKC/NF-κB-dependent manner (Table 1) [82]. In a mouse model of liver fibrosis, Xia et al. showed that S100A6 accelerated liver fibrosis through the activation of ERK in a RAGE-dependent manner, again suggesting the potential role of S100A6/RAGE in inflammation (Table 1) [83].

Among the S100 proteins, S100A8, S100A9, or the S100A8/A9 heterodimer are abundantly secreted by neutrophils, activated monocytes, and macrophages, and constitute about half of the total amount of cytosolic proteins of granulocytes [183,184]. They stimulate the transendothelial migration of leukocytes and the production of pro-inflammatory cytokines [86,182]. S100A8/A9 has also been shown to be secreted from eosinophils after stimulation with granulocyte macrophage colony-stimulating factor (GM-CSF) and IL-5 [185].

These S100 proteins are found to be elevated in the serum, synovial fluids, atherosclerotic plaques, sputum, stool, or blood/plasma of patients suffering from chronic inflammatory diseases, such as arthritis, chronic inflammatory lung and bowel diseases, or cardiovascular diseases, and are found at high levels in the microenvironment of tumors [186,187,188,189,190,191,192,193,194,195,196]. The two main receptors for S100A8/A9 are RAGE [197,198] and TLR4 [199,200]. Akari et al. recently showed that S100A8/A9 contributed to increased fibrosis in the lungs of patients with pulmonary fibrosis, in a RAGE-dependent manner [85]. The authors showed that S100A8/A9 positively regulated the proliferation of fibroblasts, as well as their differentiation into myofibroblasts, thereby resulting in higher collagen and pro-inflammatory cytokine expression. More recently, in a mouse model of sepsis, neutrophils expressing high levels of S100A8/A9 were shown to mediate mitochondrial dysfunction and the resulting cell death of endothelial cells, through the release of S100A8/A9 [87], demonstrating the important role that these S100 proteins play in inflammation. In their study, Wang et al. did not indicate if RAGE was involved in mediating the effect of S100A8/A9 [87]. In cardiac tissues, S100A8/A9 released from neutrophils was shown to interact with RAGE on cardiac fibroblasts, resulting in the activation of NF-κB. The activation of RAGE with S100A8/A9 also promoted the migration of monocytes and cardiac fibroblasts, contributing to inflammation-induced cardiac injury (Table 1) [55].

Another S100 protein with a role in inflammation is S100A10, which has been shown to help recruit macrophages by serving as a plasminogen receptor, resulting in the generation of plasmin-dependent proteolysis of the extracellular matrix, and facilitating the migration of macrophages to the site of inflammation or injury [201,202].

S100A12 is another S100 protein with inflammatory roles. S100A12 is primarily expressed by granulocytes (neutrophils), has chemotactic properties, activates inflammatory cells, and stimulates the upregulation of adhesion molecules in endothelial cells [84,203]. S100A12 stimulates the degranulation of mast cells and the synthesis of pro-inflammatory cytokines by these cells [88]. The degranulation of mast cells and the production of IL-6 and IL-8 were shown to be partly reduced in the presence of a RAGE inhibitor, suggesting a role of the RAGE signaling pathway in this process (Table 1) [88]. RAGE activation by neutrophil-released S100A12 in normal human bronchial epithelial cells resulted in the increased secretion of mucin 5AC (MUC5AC), the primary mucin found in airway mucus [56]. Another indication of the role of S100A12 in inflammation is its high level found in tissues and fluids from patients with inflammatory disorders, including Kawasaki’s disease, rheumatoid and psoriatic arthritis, inflammatory bowel disease, and allergic asthma [204,205].

One S100 member that also plays a role in inflammatory diseases is S100B (reviewed in [206]). S100B is expressed at high levels in the brain, mainly astrocytes, a subtype of glial cells [89]. S100B has both a reparative and regenerative function in the brain. In response to injury and at low concentrations, S100B has a reparative function and promotes neuronal survival. However, high levels of S100B, which can be the result of activated glial cells, can lead to increased damage from neuroinflammation and neuronal dysfunction [67,91,207,208]. Recently, in a model of cerebral ischemia, Zhou et al. showed that stimulation of murine brain-derived primary microglial cells with S100B triggers the M1 polarization of the cells [93]. S100B levels are enhanced during astrogliosis or inflammation of astrocytes [209]. This process is observed after brain insults and is associated with neuronal inflammation, as observed in Alzheimer’s disease (AD), amyotrophic lateral sclerosis, or Parkinson’s disease [209]. In a transgenic mouse model of Alzheimer’s disease, overexpression of S100B resulted in increased Aβ deposits, enhanced astrocytosis and microgliosis, and higher levels of pro-inflammatory cytokines, when compared to control mice [210]. Correspondingly, in a different AD mouse model, ablation of S100B resulted in reduced astrocytosis and plaque levels in the animals [211]. In a chronic experimental autoimmune encephalomyelitis mouse model of multiple sclerosis, deletion of S100B resulted in reduced motor impairments, myelin degradation, and glial reactivity, as well as reduced expression of the pro-inflammatory cytokines TNF-α and IL-1β, compared to the control animals (Table 1) [212]. Although S100B is considered mainly as a biomarker in neuroinflammatory disorders, experimental evidence also suggests a role of S100B in inflammatory diseases of the gut, such as in inflammatory bowel disease [206,213].

As mentioned earlier in this paragraph, S100B can have neuroprotective and neurotoxic effects. Recent studies have recently unraveled new protective functions of S100B in the brain. Cristovao et al. showed that S100B could act as a chaperone that prevents Aβ aggregation and toxicity at early stages of AD [214]. In a later study, the same research group showed that S100B also acted as a chaperone with the tau protein [215]. The tau protein interacts with and stabilizes microtubules in cells. Aggregation of the tau protein results in protein dysfunction, and is associated with a large group of diseases, called tauopathies, that include frontotemporal dementia, Parkinson’s disease, and AD [215]. Interestingly, Esposito et al. previously showed that at micromolar concentrations, S100B could stimulate the phosphorylation of the tau protein, and further promote neuroinflammation, in a RAGE-dependent manner, through the activation of JNK, the nuclear factors AP-1 and c-Jun, and the activation of Dickopff-1 and glycogen synthase kinase (GSK)3β (Table 1) [92]. These data further demonstrate the two opposite functions that S100B can have in the brain, depending on its concentration.

## 5. RAGE Expression in Immune Cells and Its Role in Immune Responses

### 5.1. RAGE in Dendritic Cells

**Figure 3:**
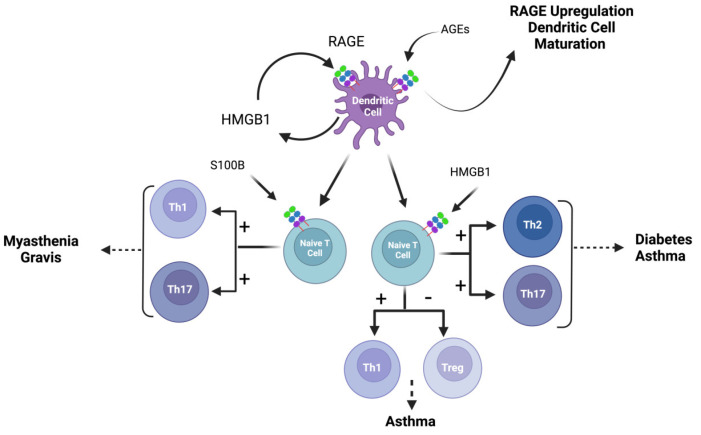
RAGE and its ligands in dendritic cells (DC) and T cells. AGEs stimulate the maturation of DCs and enhance their abilities to stimulate T cells and upregulate RAGE expression [62,63]. HMGB1 released from DCs acts on RAGE to promote functional polarization of T cells [53]. RAGE activation by HMGB1 in DCs results in the differentiation of naive T cells into Th2 and Th17 [54]. RAGE activation by HMGB1 can also result in the imbalance between Treg and Th17 in asthma [57]. RAGE activation by S100B results in the differentiation of naive T cells into Th1 and Th17 in a mouse model of Myasthena Gravis [220].

Dendritic cells play a crucial role in linking the innate and adaptive immune response by presenting antigens and assisting in the activation of T cells. The mobilization of dendritic cells from peripheral tissues is important for T-cell-dependent immune responses, as dendritic cells must interact with naive T cells in lymph nodes [216]. RAGE plays a critical role in the maturation and mobilization of dendritic cells. In an in vivo study, Manfredi et al. showed that RAGE-deficient dendritic cells (DCs) failed to mobilize to lymph nodes when injected into mice [217]. Similarly, necrotic fibroblasts from HMGB1^−/−^ mice were not able to activate DCs when compared to necrotic fibroblasts from wild-type mice [218], confirming studies by others indicating that HMGB1 is an immunostimulatory signal inducing DC maturation (Figure 3) [219]. In a later study, Dumitriu et al. showed that HMGB1 released from DCs acted on DCs through RAGE and NF-κB, and that secreted HMGB1 was necessary for the clonal expansion, survival, and functional polarization of naive T cells [53]. These studies demonstrate the critical role of HMGB1 and the HMGB1/RAGE signaling pathway in stimulating the maturation of dendritic cells.

### 5.2. RAGE in T Cells

An important function of DCs is to interact with and activate T cells in lymph nodes. As described in the previous section, HMGB1 secreted from DCs is an important signal molecule for T cells [53]. In a mouse model of asthma, Li et al. showed that activation of RAGE by HMGB1 in DCs resulted in the differentiation of naive T cells into type 2 and 17 helper T cells (Th2 and Th17)) through the activation of the RAGE/NF-κB signal pathway in the T cells (Figure 3) [54]. In the same study, the authors showed that HMGB1 could induces the polarization of naive T cells into Th2 and Th17 by direct interaction with T cells [54].

The expression of RAGE by CD4^+^ and CD8^+^ T cells was first reported by Chen et al. in a diabetic mouse model [24]. In a later study, Akirav et al. showed that CD4^+^ and CD8^+^ T cells isolated from Type 1 (T1D) and Type 2 (T2D) Diabetes patients express RAGE, whereas resting T cells from healthy patients do not express RAGE. In this study, RAGE was found in the endosomes of T cells, and the reason for this cellular location is currently unknown [221]. T cells isolated from patients that had a great risk of later developing diabetes were shown to express RAGE before these patients presented clinical disease. Interestingly, RAGE-positive cells were not found in patients with rheumatoid arthritis or Sjogren’s syndrome, which led the authors to conclude that the expression of RAGE in T cells was not a general feature of autoimmunity or inflammatory conditions [221]. By using a proximity ligation assay, Durning et al. showed that in T cells, endosomal RAGE bind to extracellular HMGB1 through a mechanism that has not been yet fully elucidated [222]. In addition, when incubated with HMGB1, T cells originating from T1D patients generated higher levels of interferon gamma and IL-6 than healthy control cells, suggesting HMGB1-dependent activation of these cells [222]. RAGE-positive T cells also showed increased survival in nutrient deprivation conditions compared to RAGE-negative T cells [222]. A later study showed that RAGE expression in human T cells was associated with an activated signaling cascade, characterized by increased phosphorylation of ERK, Zap70, and MEK [223].

Although the study by Akirav et al. showed that RAGE-positive cells are not found in patients with rheumatoid arthritis or Sjogren’s syndrome [221], there is evidence that the binding of different ligands to RAGE can influence T cell differentiation and contribute to the pathogenesis of autoimmune diseases. Mu et al. reported that when T cells isolated from a mice model of myasthenia gravis were stimulated with S100B, the interaction of S100B and RAGE shifted T cell differentiation to Th1 and Th17, a T helper cell profile associated with disease pathogenesis (Figure 3) [220]. In a different model, reducing the binding of HMGB1 to RAGE was shown to alleviate T regulatory (Treg)/Th17 immune imbalance, and block allergic rhinitis from developing into asthma (Figure 3) [57]. In another study, RAGE expression on Treg and CD8^+^ cells was found to be two-fold higher in patients with severe psoriasis compared to healthy individuals. An increase in serum levels of HMGB1 was also seen in these patients, suggesting a role of HMGB1/RAGE in the progression of the autoimmune disease [224].

### 5.3. RAGE in Monocytes and Macrophages

Monocytes and macrophages originate from a common myeloid precursor, and have key functions in innate and adaptive immunity by maintaining homeostasis, regulating inflammation, and activating other immune cells such as T cells through their activities as antigen-presenting cells [225,226,227,228,229]. Inflammation prompts circulating monocytes to rapidly invade affected tissues and to differentiate from macrophages. Macrophages are found in every tissue of the body and can ingest pathogens or dead cells via phagocytosis. Macrophages can be activated following interactions with pathogens (non-sterile inflammation) or by DAMPs (sterile inflammation), and can release cytokines to recruit other immune cells to sites of injury. Macrophages are typically classified into two subtypes (M1 and M2) based on their mechanisms of activation and immune-regulating activities. M1 macrophages are classically activated by IFN-γ or lipopolysaccharide (LPS), and present high phagocytic activity, release high levels of pro-inflammatory cytokines (TNF-α, IL-1α, IL1β, IL-6, IL-12 and IL-23), and produce reactive oxygen species against invading pathogens. M2 macrophages are activated by the cytokines IL-4, IL-10, or IL-13, present high phagocytic activity, and are primarily associated with tissue repair and wound healing. M2 macrophages can be further separated into sub-populations based on their cell surface molecule expression patterns and cytokine expression profiles [58,225,226,227,228,229,230,231,232]. Monocytes and macrophages express RAGE on their surfaces [233] (reviewed in [234]). In the following paragraphs, we will give examples of studies demonstrating the role of RAGE in modulating the activation and differentiation of macrophages in different biological situations.

#### 5.3.1. RAGE and Macrophage Polarization in Diabetic Neuropathy

**Figure 4:**
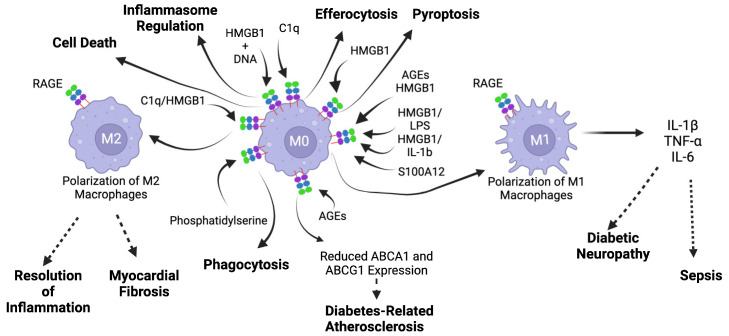
RAGE and its ligands in macrophages. RAGE activation by AGEs stimulates the M2 to M1 polarization of macrophages and plays a role in the progression of diabetic polyneuropathy [235,236]. LPS/RAGE plays a role in sepsis though the secretion of pro-inflammatory cytokines (IL-1*β*, IL-6, TNF-*α*) [239]. S100A12/RAGE stimulates the migration of mononuclear phagocytes, the secretion of pro-inflammatory cytokines (IL-1*β* and TNF-*α*) by macrophages, and the proliferation of PMBC [4]. AGE/RAGE activation results in reduced expression of the ABCA1 and ABCG1 cholesterol transporters in diabetic macrophages [240]. HMGB1/LPS complexes stimulate macrophages [65]. HMGB1/nucleic acid complexes regulate the activation and termination of inflammasomes in a RAGE-dependent manner, and can also trigger cell death [71,72]. RAGE interaction with PS in macrophages stimulates phagocytosis [141,142]. HMGB1 promotes inflammation when in a complex with LPS, IL-1*β*, or nucleic acids [17,65,135,241]. RAGE promotes the internalization of HMGB1/LPS and the delivery of LPS intracellular caspase 11, resulting in pyroptosis [70]. C1q interacts with RAGE and enhances phagocytosis [14]. In a complex with HMGB1, C1q promotes the resolution of inflammation [73]. RAGE mediates HMGB1-/LPS-dependent pyroptosis [68,70]. RAGE mediates the secretion of (IL-1*β*, IL-6, TNF-*α*) in macrophages in a mouse model of obese mice [242]. RAGE mediates myocardial fibrosis by recruiting M2 macrophages [231].

Many studies suggest that RAGE is an important determinant of the polarity of macrophages [235,236,237,238]. A study by Jin et al. showed that by binding to and activating RAGE at the surface of macrophages, AGEs enhanced M2 to M1 polarization through the activation of NF-κB (Figure 4) [236]. In a mouse model of diabetes, Juranek et al. observed that a higher number of M2-polarized macrophages had infiltrated the diabetic nerve tissues in RAGE null mice than in WT animals [237]. In their in vitro study, the authors also showed that macrophages derived from WT mice bone marrow and treated with AGEs showed increased levels of M1 expression markers compared to non-treated macrophages, and that this change in expression levels was RAGE-dependent (Figure 4) [237].

In a different mouse model of diabetic neuropathy, it was found that RAGE-null mice showed greater numbers of M2 macrophages than control RAGE-positive mice. The progression of diabetic polyneuropathy is associated with an increase in inflammatory cells and cytokine expression in the peripheral nerves, and pro-inflammatory changes in the bone marrow. Bone marrow transplantation from RAGE-null mice to diabetic mice had restorative effects on peripheral nerve deficits, which led the authors to conclude that RAGE controls the differentiation of macrophages into the M1 pro-inflammatory phenotype and plays a role in the progression of diabetic polyneuropathy (Figure 4) [235].

#### 5.3.2. RAGE and Microglia in Alzheimer’s Disease

**Figure 5:**
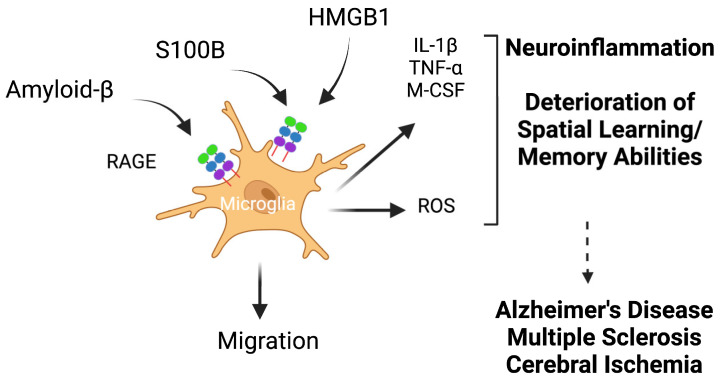
RAGE and its ligands in microglia. Amyloid *β* mediates the production of the pro-inflammatory cytokines IL-1*β* and TNF-*α* by microglia in a RAGE-dependent manner, leading to neuroinflammation [78]. S100B stimulates the polarization of microglial cells into M1 in mouse models of cerebral ischemia and multiple sclerosis [93,212]. In mouse models of brain ischemia, HMGB1 activates microglial RAGE, resulting in the expression of the pro-inflammatory cytokines IL-1*β*, TNF-*α*, and Il-6 [244,245].

In the context of Alzheimer’s disease (AD), microglia, which are the resident macrophages of the brain, play an important role in neuroinflammation. RAGE is expressed in microglia and triggers microglial activation following its interaction with amyloid β peptides, an important RAGE ligand [12,243]. Using transgenic mouse models of AD, with the overexpression of microglial RAGE and the amyloid precursor protein (APP), Fang et al. showed that amyloid β was in part responsible for the increased production of the pro-inflammatory cytokines IL-1β and TNF-α by the microglia in a RAGE-dependent manner (Figure 5) [78]. In mouse models of brain ischemia, Liesz et al. also showed that during the acute phase following a stroke, HMGB1 was released and activated RAGE in macrophages and dendritic cells, resulting in increased secretion of the pro-inflammatory cytokines IL-1β, TNF-α, and Il-6 (Figure 5) [244], in agreement with earlier studies [245].

#### 5.3.3. Macrophage RAGE in Pancreatic Tumor Microenvironment

Irreversible electroporation (IRE) is a type of ablation therapy that has been explored for the treatment of pancreatic cancer. IRE uses high-voltage, low-energy electric pulses to induce cell death (reviewed in [246]). Studies have shown that IRE treatment of pancreatic cancer and melanoma cells resulted in the release of the DAMP molecules HMGB1 and ATP in the culture media, resulting in immunologic cell death [58,247]. The exposure of macrophages with the conditioned media from tumors treated by IRE resulted in the differentiation of the macrophages into the M1 subtypes [58]. In these M1-differentiated macrophages, HMGB1 was shown to increase RAGE expression, RAGE activation through the MAPK-ERK pathway, and the additional release of HMGB1 in the media [58].

#### 5.3.4. RAGE and Macrophages in Adipose Tissues

In a mouse model of obesity, RAGE deletion reduced adipose tissue inflammation and improved glucose and insulin tolerance [242]. RAGE deficiency also reduced the recruitment of macrophages to epidymal adipose tissue. In these mice, the levels of the pro-inflammatory cytokines IL-1β, IL-6, TNF-α, and monocyte chemoattractant protein-1 (MCP-1) secreted from infiltrated macrophages were reduced when compared to control mice (Figure 4) [242].

#### 5.3.5. RAGE in Sepsis

There is experimental evidence that RAGE regulates sepsis. In a mouse model of LPS-induced sepsis, lower serum levels of pro-inflammatory cytokines (IL-1β, IL-6, TNF-α) were observed in RAGE knock-out (−/−) mice than in the control wild-type RAGE^+/+^ animals, following an LPS insult (Figure 4). RAGE^−/−^ mice also exhibited increased survival rates compared to RAGE^+/+^ animals. In addition, the level of pro-inflammatory cytokines was reduced following treatment with the soluble form of the RAGE receptor (sRAGE). Moreover, LPS was found to induce RAGE-dependent secretion of TNF-α and the activation of NF-κB in isolated peritoneal macrophages [239]. In a different mouse model of sepsis, consisting of cecal ligation and puncture (CLP), RAGE^−/−^ mice also had better survival than their counterpart RAGE^+/+^ animals [23,248]. Interestingly, the treatment of septic RAGE^−/−^ mice with sRAGE also protected against septic shock, suggesting that the beneficial effects of sRAGE were not caused by preventing ligand engagement of cell surface RAGE, and that other receptors were involved [23]. However, Prantner et al. recently showed that RAGE deletion did not provide a survival advantage in mice after peritoneal injection of LPS [249], suggesting that additional studies need to be performed in this area to clarify the role of RAGE during sepsis.

#### 5.3.6. RAGE and Cholesterol Efflux in Macrophages

An important property of macrophages is to modulate the efflux of cholesterol through the modulation of the expression of two ATP binding cassette transporters: ABCA1 and ABCG1. Defects in reverse cholesterol transport, a mechanism by which the body removes excess cholesterol from peripheral tissues, have been demonstrated in human diabetic patients and in mouse models of diabetes, resulting in higher risks of cardiovascular disease through increased atherosclerotic lesions or plaques (reviewed in [250]). Studies have shown a partially RAGE-dependent reduction in the expression of the ABCA1 and ABCG1 cholesterol transporters in animal models of diabetes (Figure 4) [240]. A larger reduction in ABCG1 transporter expression was observed after stimulation of RAGE by its CML-AGE ligand. Cholesterol efflux from macrophages was improved in RAGE-deleted diabetic macrophages compared to RAGE-expressing diabetic macrophages [240].

#### 5.3.7. Macrophage RAGE in Atherosclerotic Plaques

Macrophages are present in high levels in atherosclerotic plaques [251], and RAGE expression has been found to be increased in the atherosclerotic plaques of diabetic patients and associated with enhanced inflammatory reactions [252]. In a post-mortem analysis of atherosclerotic plaques following sudden death, RAGE and its S100A12 ligand were found at higher levels in the plaques of diabetic individuals than non-diabetic individuals [253]. The role of RAGE in diabetic atherosclerosis was confirmed in multiple animal studies [254,255,256].

#### 5.3.8. RAGE in Myocardial Fibrosis

Macrophages are important contributors to myocardial fibrosis (MC), which is a type of fibrosis observed in the heart in certain cardiovascular pathologies, and which can lead to heart failure. In MC, the role of the M2 pro-fibrotic macrophages is to stimulate the differentiation of fibroblasts into myofibroblasts, resulting in the excessive accumulation of extracellular matrix proteins and myocardial dysfunction [231]. The study of He et al. showed that RAGE deficiency in macrophages resulted in the decreased infiltration of M2 profibrotic macrophages in the heart tissue in experiments mimicking cardiac pressure overload. This resulted in a reduced interstitial fibrosis and cardiac dysfunction, suggesting that RAGE plays a crucial role in myocardial fibrosis by recruiting M2 macrophages (Figure 4) [231].

#### 5.3.9. RAGE in Lung Fibrosis

Among all tissues, RAGE is expressed at the highest level in the lungs, and more specifically in the basal membrane of type 1 alveolar (AT1) cells [257,258]. Studies have shown that RAGE^−/−^ mice develop lung fibrosis when they age, suggesting that RAGE plays a role in lung fibrosis [259]. However, controversial and opposite outcomes in different animal models of lung fibrosis later led to the conclusion that the role of RAGE in lung fibrosis is complex and depends on the experimental conditions used to model sepsis [3,260,261,262,263]. A possible mechanism of RAGE in lung epithelial cells was provided by Queisser et al. [264]. These authors showed that RAGE was significantly downregulated in lung homogenate and alveolar epithelial isolated from patients with idiopathic pulmonary fibrosis, as well as from a bleomycin-induced mouse model of lung fibrosis. The same authors also showed that RAGE deletion in lung epithelial cells and primary lung fibroblasts resulted in decreased cell adhesion and increased cell migration, associating RAGE with specific functions in these cell types [264].

### 5.4. RAGE in Granulocytes

#### 5.4.1. RAGE in Neutrophils

RAGE also plays a crucial role in regulating granulocytes, the most abundant innate immune cells in the body. Granulocytes are named after their high content of granules that contain antimicrobial and cytotoxic molecules, which are released upon activation. The subtypes of granulocytes include neutrophils, eosinophils, and basophils, which are classified according to their histological staining characteristics [265]. Neutrophils form the most abundant subtype of granulocytes, and are the first leukocytes recruited from the bloodstream to sites of infection or injury. Neutrophils employ a unique antimicrobial mechanism consisting of the formation of neutrophil extracellular traps (NETs), which are web-like structures composed of cytosolic and granule proteins built on a scaffold of decondensed chromatin [265,266].

**Figure 6:**
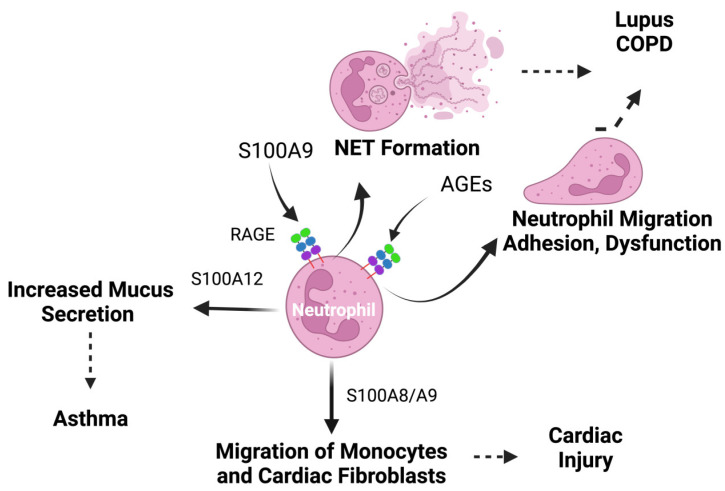
RAGE and its ligands in neutrophils. AGE/RAGE mediates neutrophil dysfunction, adhesion, and migration [267,268,269]. S100A9/RAGE elicits NET formation in neutrophils [270]. S100A12 released from neutrophils activates RAGE in human bronchial epithelial cells, resulting in mucous metaplasia [56]. S100A8/A9 released from neutrophils interacts with RAGE on cardiac fibroblasts, resulting in the migration of monocytes and cardiac fibroblasts and inflammation-induced cardiac injury [55].

Neutrophils not only secrete RAGE ligands, but their activity can also be modulated by RAGE ligands. Indeed, the activation of RAGE by AGEs has been shown to mediate neutrophil dysfunction (Figure 6) [267]. In a different study, RAGE was found to mediate neutrophil adhesion and migration onto the glycated extracellular matrix (Figure 6) [268], as well as neutrophil migration across the intestinal epithelium [269]. Tatsiy et al. showed that S100A9 activates neutrophils upon binding with RAGE, rapidly promoting ERK phosphorylation and eliciting NET formation (Figure 6) [270].

RAGE has also been shown to regulate neutrophils in a mouse model of lupus erythematosus. In this model, RAGE deficiency was found to significantly reduce neutrophil infiltration and NET formation in the glomerulus, resulting in markedly improved renal pathological scores [266]. In a mouse model of chronic obstructive pulmonary disorder (COPD), RAGE expression correlated with airway neutrophilia and airway hyperresponsiveness following insults with cigarette smoke (Figure 6) [271]. In a model of asthmatic neutrophil-dominant airway inflammation in mice, RAGE expression was associated with elevated levels of Th1/Th17 cytokines, increased NETs, and sustained neutrophil accumulation compared to RAGE-null mice [59]. In airway epithelial cells, treatment with RAGE inhibitors not only diminished the expression of RAGE and its ligands, but also reduced neutrophil-predominant airway inflammation and injury, decreased levels of IL-6, IL-1β, and TNF-α in BALF, and alleviated increased alveolar–capillary permeability and pulmonary edema [272]. All of these data are strong arguments to suggest that RAGE promotes inflammation in neutrophil-dominant airway inflammation.

#### 5.4.2. RAGE in Eosinophils and in Eosinophilic Asthma

**Figure 7:**
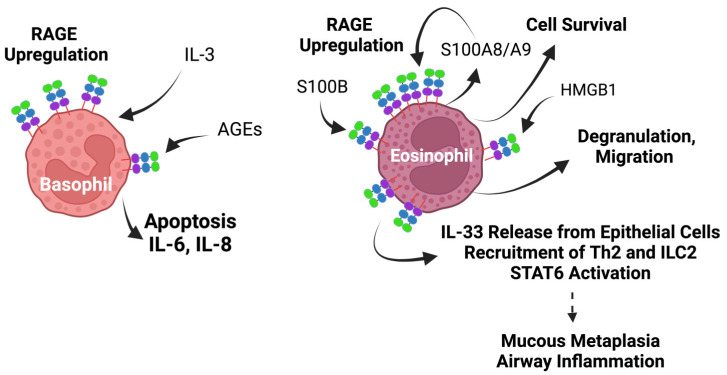
RAGE and its ligands in eosinophils and basophils. S100B/RAGE stimulates the secretion of S100A8/A9, the upregulation of RAGE, and cell survival [185]. RAGE mediates IL-33 release from epithelial cells, and the recruitment of Th2 and ILC2 cells to the site of inflammation in inflamed airways. In addition, RAGE mediates the activation of the signal transducer and activator of transcription 6 (STAT6), resulting in airway inflammation and mucus metaplasia [274,275]. S100A8/A9 acts in an autocrine manner to further amplify RAGE signaling [275]. HMGB1 stimulates the degranulation and migration of eosinophils [273].

Eosinophils stain positive for RAGE, both intracellularly and at the cell surface, and have been demonstrated to be activated by HMGB1 in degranulation and migration assays (Figure 7) [273]. Eosinophils isolated from human blood have been shown to respond to S100B by upregulating RAGE expression, as well as S100A8 and S100A9 secretion (Figure 7) [185]. In addition, eosinophil activation by S100B has been shown to result in PKδ activation, increased expression of CD11b on the surface of eosinophils, and enhanced survival [185].

During asthmatic reactions, eosinophils are key mediators of type Th2 inflammatory responses which are initiated by the release of epithelial-derived cytokines, such as IL-33. Th2 and type 2 innate lymphoid cells (ILC2) together release large amounts of cytokines, including IL-4, IL-5, and IL-13. IL-5 promotes the recruitment of eosinophils, their activation, proliferation, and survival, whereas IL-4 and IL-13 stimulate goblet cell hyperplasia, mucus hypersecretion, chemokine expression, and airway hyperresponsiveness (Figure 7).

The role of RAGE in the pathology of Th2 eosinophilic airway inflammation is supported by many animal studies using RAGE^−/−^ mice [275,276,277,278,279]. There is strong evidence that in Th2 eosinophilic asthma, RAGE is required for the release of allergen-mediated IL-33 from epithelial cells, as well as for the recruitment of Th2 and ILC2 cells to the site of inflammation. In addition, RAGE is also required for the IL-4-/IL-13-dependent phosphorylation of the signal transducer and activator of transcription 6 (STAT6), resulting in chemokine production, airway inflammation, and mucus metaplasia, therefore contributing to the severity of asthma (Figure 7) [274,275]. An outcome of RAGE activation is the downstream secretion of S100A8/A9, that can act in an autocrine manner to further amplify RAGE signaling [275]. Studies have indeed shown that S100A8/A9 levels correlate with asthma severity in human subjects [280]. These observations provide additional evidence for the role of RAGE and its S100A8/A9 ligands, not only in neutrophil-dominant airway inflammation, but also in eosinophilic asthma (Figure 7).

The activation of RAGE by its ligands has been shown to activate basophils. In these cells, AGEs stimulated the apoptosis of basophils and dose-dependently increased the secretion of IL-6 and IL-8 [281]. The stimulation of basophils with IL-3 has also been shown to increase the levels of RAGE (Figure 7).

## 6. Conclusions

The role of RAGE in inflammation is highly complex and cannot be described in a single, all-encompassing model. RAGE is expressed by most immune cells (dendritic cells, T cells, monocytes, macrophages, granulocytes, eosinophiles) and is frequently, but not always, upregulated in pathological conditions [1,5,6]. The predominant form of RAGE is the plasma-membrane-bound full-length form of RAGE. However, soluble forms of the RAGE ectodomain, either generated by proteolytic clipping or by alternative splicing, are also (patho)physiologically relevant, and are generally considered to impede RAGE signaling [35,36,37,38].

A very difficult fact to dissect is that RAGE activation involves many inflammation-relevant ligands, which act through non-RAGE receptors as well, in particular the S100 proteins (S100A2, 100A4, S100A6, S100A8, S100A9, S100A12, S100B), advanced glycation end-products, complement protein C1q, amyloid peptides, and HMGB1 [7,8,9,10,11,12,13,14,15,16,17]. RAGE ligands act simultaneously on immune and non-immune cells under physiological conditions. At the same time, RAGE activation can lead to the upregulation of RAGE expression through a positive feed-forward signaling cascade involving NF-κB, and also increases the expression and secretion of many RAGE ligands. In addition, RAGE activation can lead to the upregulation of additional pro-inflammatory proteins. As a consequence, immune cells are recruited to the sites of RAGE activation and establish an inflammatory environment [39,44]. An additional level of complexity is added by the observation that the cellular consequence of RAGE activation can be concentration-dependent, as reported with S100B. Low levels of S100B have neurotrophic effects, while high concentrations of S100B show neurotoxic effects [208].

Besides functioning as a cell surface receptor, RAGE plays a role in the endocytotic process of RAGE ligands inside the cell. This has been demonstrated for AGEs, amyloid peptides, and HMGB1. Sometimes, these ligands can carry additional molecules inside the cell, as has been demonstrated for the internalization of the RAGE/HMGB1/LPS complex [17,65,70,135,241] and, most recently, with a bacterial toxin [134] that can result in pyroptosis. RAGE also has the ability to recruit co-receptors, such as Mac1 and TLR4, leading to additional intracellular signaling pathways [75,282]. In certain cell types, such as T cells, RAGE is observed predominantly intracellularly, suggesting that RAGE or RAGE/ligand complexes can have additional biological roles beyond recognizing extracellular ligands and initiating intracellular signaling [31,221].

Studies with RAGE^−/−^ animals have clearly established the relevance of RAGE to a range of inflammatory disease conditions [3,23,59,217,218,231,237,239,240,242,248,254,256,260,261,262,263,266,275,276,277,278,279]. This suggests that the pharmacological inhibition of RAGE could be a promising approach to improve the treatment of a broad spectrum of health issues, ranging from diabetic complications to neurodegeneration and heart failure. The prospect of developing ligand-specific RAGE inhibitors and their pharmacological potential is an exciting area of research, and may lead to better therapies in the future.

## Figures and Tables

**Table 1:** Effect of selected RAGE ligands on immune cells and other cells relevant to inflammation.

Ligand	Cell/Animal Model Used	Effect on Cells	Disease/Disease Model	References
AGE-β2-microglobulin	Human mononuclear phagocytes	Stimulates RAGE-dependent production of tumor necrosis factor alpha (TNF-α), oxidative stress, and chemotaxis	Dialysis-related amyloidosis	[61]
AGE-ovalbumin	Human immature dendritic cells (DC)	Enhances RAGE expression and NF-kB translocation to the nucleus Enhances IL-6 production	Food allergy	[62]
AGE-BSA	Monocyte-derived DCs	Enhances RAGE expression Promotes RAGE- and c-Jun N-terminal kinase (JNK)-dependent DC maturation Enhances the ability of DCs to activate T cells	Atherosclerosis	[63]
AGE-BSA	Rat peritoneal mast cells	Triggers RAGE-dependent exocytosis, histamine release and reactive oxygen species (ROS) production	Chronic inflammatory diseases	[64]
HMGB1/lipopolysaccharide (LPS)	Murine peritoneal macrophages	Triggers TNF-α and interleukin -6 (IL-6) secretion RAGE-dependent activation of p38 and NF-kB	Inflammation	[65]
HMGB1/nucleic acid	Murine DCs	Stimulates RAGE- and Toll like receptor -9 (TLR-9)-dependent cytokine production	Lupus	[66]
HMGB1	Rat brain cortical neuron	Stimulates RAGE-dependent neurite outgrowth	Neuronal function	[67]
HMGB1	Murine macrophage	Triggers RAGE-dependent macrophage and monocyte pyroptosis	Endotoxemia	[68]
HMGB1/LPS	Human monocyte	Enhances TNF-α production	Gram–bacteria induced sepsis	[69]
HMGB1/LPS	Murine peritoneal macrophages	RAGE-mediated HMGB1/LPS internalization HMGB1 enables LPS to activate intracellular caspase 11 and triggers pyroptosis.	Sepsis	[70]
Nucleic acid/HMGB1	Human monocytic cell lines	Stimulates both RAGE-dependent activation and inhibition of inflammasome	Inflammation	[71]
Nucleic acid/HMGB1	Murine RAW264.7 macrophages	Enhances RAGE-dependent activation of AKT, TNF-α release and cell death	Inflammation	[72]
C1q	U937-derived phagocytes	Triggers RAGE-dependent phagocytosis	Innate and adaptive immune response	[14]
C1q/HMGB1	Peripheral blood monocytes	C1q inhibits HMGB1-induced monocyte activation C1q inhibits RAGE-dependent HMGB1 internalization C1q bridges RAGE and LAIR Promotes resolution of inflammation and the expression of resolvin D1 and D2 and lipoxin A4		[73,74]
MAC-1	Mouse model	Stimulates RAGE-dependent leukocyte recruitment RAGE/MAC-1 interaction enhanced in the presence of S100B	Mouse model of acute peritonitis	[75]
MAC-1/HMGB1	Mouse model	HMGB1 promotes RAGE-/MAC-1-dependent neutrophil recruitment HMGB1 enhances RAGE/MAC-1 interaction HMGB1 triggers RAGE- and MAC-1-dependent NF-κB activation		[76]
Amyloid β (Aβ)	Microglia	Stimulates RAGE-dependent expression of M-CSF	Alzheimer’s Disease	[77]
Amyloid β (Aβ)	Neurons	Triggers RAGE- and NF-κB-dependent expression of M-CSF	Alzheimer’s Disease	[12]
Amyloid β (Aβ)	Brain tissue	Stimulates IL-1β and TNF-α production Enhances infiltration of microglia and astrocytes and Aβ accumulation Accelerates deterioration of spatial learning/memory abilities	Alzheimer’s Disease	[78]
Amyloid β (Aβ)	Human endothelial cells, neuronal cells, and microglia	Enhances RAGE-dependent oxidant stress and NF-κB activation in endothelial cells and neurons Stimulates RAGE-dependent migration of microglia and TNF-α expression	Alzheimer’s disease	[79]
S100A4	Mouse model	Mediates macrophage recruitment and chemotaxis	Inflammation	[80]
S100A4	Human peripheral blood samples from patients with rheumatoid arthritis	Stimulates TNF-α, IL-1β, and IL-6 secretion	Rheumatoid Arthritis	[81]
S100A6	THP-1 monocytes	The TgSAG1 protein from T. gondii promotes the expression of TNF-α in a S100A6/vimentin- and PKC/NF-κB-dependent manner	Toxoplasma gondii infection	[82]
S100A6	Mouse model of liver fibrosis	S100A6 triggers RAGE-dependent ERK phosphorylation and accelerates liver fibrosis	Liver fibrosis	[83]
S100A8, A9	In vitro	Promotes leukocyte recruitment S100A9 induces MAC-1 expression	Inflammation	[84]
S100A8/A9	Murine fibroblasts	Stimulates RAGE-dependent fibroblast proliferation and differentiation Increases collagen production Promotes RAGE-dependent NF-κB activity	Lung fibrosis	[85]
S100A8/A9	Human macrophages	Stimulates TNFα, IL-1β, and IL-6 production	Inflammation	[86]
S100A8/A9	Murine endothelial cells	Stimulates cell death (PANapoptosis) of endothelial cells	Mouse model of sepsis	[87]
S100A8/A9	Cardiac fibroblasts	Triggers RAGE-dependent NF-kB activation Stimulates monocytes and cardiac fibroblasts migration	Inflammation-induced cardiac injury	[55]
S100A12	Human monocytes	Stimulates TNF-α and IL-1β production Increases adhesion receptor expression in endothelial cells	Inflammation	[4]
S100A12	Cord-blood-derived mast cells	Stimulates degranulation of mast cells Enhances RAGE-dependent TNF-α, IL-6, IL-8, MCP-1, and MIP-1β secretion	Inflammation	[88]
S100A12	Human bronchial epithelial cells	Stimulates RAGE-dependent secretion of the MUC5AC mucin	Lung inflammation	[56].
S100B	neurons	Neurotrophic effect at low concentration Neurotoxic effect at high concentration	Neuroinflammation	[89]
S100B	Rat astrocyte	Stimulates nitric oxide (NO) production	Neuroinflammation	[90]
S100B	RAGE-transfected N18 neuroblastoma cells on HMGB1-coated plates	Stimulates RAGE- and NF-κB-dependent neurite outgrowth at low concentration but triggers cell apotosis at high concentration	Neuroinflammation	[91]
S100B	Neuronal stem cells	Stimulates RAGE-dependent tau hyperphosphorylation through increases in JNK, AP-1/c-Jun, Dickopff-1, and GSK3β phosphorylation	Alzheimer’s disease	[92]
S100B	Brain-derived murine primary microglial cells	Stimulates M1 polarization Triggers increases in inducible NO synthase (iNOS), TNF-α, and IL-6 Stimulates decreases in IL-10 and TGF-β production	Cerebral ischemia	[93]

## Data Availability

No new data were created or analyzed in this study.
